# M. Velpeau on Puerperal Convulsions

**Published:** 1835-07-01

**Authors:** 


### Des Convulsions chez les Femmes, pendant la Grossesse, pendant le Travail, et apres l'Accouchement. Par *A. Velpeau.* Octavo, pp. 136.

M. Velpeau very justly remarks, that the adoption of a single appellative, such as convulsion, fever, &c. to designate a train of morbid symptoms, which are neither steady nor uniform in their features, succession, causes, or effects, has induced much practical evil into the pursuits of medicine. Convulsions, like fever, are only terms, which signify the existence of certain phenomena, or train of phenomena, without any reference to the pathological states which give rise to them. True, indeed, it is, that in the present state of our knowledge, we are far, very far, from the attainment of such precision in our enquiries after the actual and essential nature of almost any malady, to justify the introduction of a purely pathological nomenclature ; and perhaps we hazard little in asserting, that such exact information can never be reached. The animal body is something more than a mere wondrous machine, consisting of visible parts and organs, whose actions, although silent, are ever active with incessant play; these actions we may, indeed, examine and investigate, tracing their effects, concatenations, and the influences which promote or disturb them; but beyond this we cannot go: we see, for example, that a muscle moves when its nerves are irritated, or when the messages of the will are sent along them; but who can tell what is the nature of the changes which take place in the state of the nerves at the time? Now, if we are so ignorant of the mysteries of a healthy function, surely we cannot be surprised that the "primum mobile" of its morbid or abnormal phenomena should be hidden from our ken.

These few remarks, although trite and common-place, are worthy of all attention whenever we are engaged in any enquiries relative to the nervous system, and more especially in our attempts to elucidate the etiology of those diseases which arise from disturbances of that system.

The truth of this must be apparent at the very threshold of our present discourse. Some of the anatomist-physicians of modern times have laboured to prove, that convulsions are always dependent upon an inflammatory, or at least a hyperemic condition of some part of the nervous system, and therefore, demand on all occasions an antiphlogistic treatment, wilfully shutting their eyes to the every-day fact, that the disease often occurs in the feeblest and most exhausted subjects, who exhibit not one symptom of sanguineous excitement, or of plethora; that they frequently supervene on profuse hæmorrhages, and other discharges; that they are benefited by stimulants, and made worse by depletives, and that the very results of post-mortem examinations, on which they wish to establish their creed, are often nugatory, and sometimes quite opposed.

The convulsions which occur in the puerperal (using that term to embrace the periods of gestation, labour, and the first week or two after delivery) state, exhibit a considerable variety in their obvious characters; sometimes they are allied to the phenomena of an hysterical or epileptic fit—at other times, they partake more of a tetanic or apoplectic diathesis; hence the practically useful division of the genus "eclampsia" into the four orders now enumerated. The convulsive movements may be either local, affecting one set of muscles, as those of the face, of one limb, &c. or general, when all the voluntary muscles, and sometimes, also, those which belong to the mixed and involuntary classes, are affected.

Partial convulsions are by no means of very frequent occurrence; unless indeed, we include under this term the obstinate vomitings which so distress some patients during gestation and labour—the violent palpitations, and other analogous symptoms. These phenomena, however, although no doubt partaking somewhat of the character of partial convulsions, are very properly excluded from the present enquiry, and demand for themselves a special consideration. The genuine partial convulsions may be either tonic or clonic; in other words, they may be either fixed, permanent, and, as it were, tetanic, or they may be rapidly recurring, with short intervals of repose, and accompanied with irregular and involuntary movements, more or less "bizarre." Not unfrequently (more especially during the first four months of pregnancy) the convulsive paroxysms are preceded by a sense of a ball, rising from the hypogastrium till it reaches the throat, well known to medical men by the name of the "globus hystericus." Although not quite connected with our subject, we may state that this symptom sometimes occurs in male patients; two well characterized cases are detailed in M. Dubois' Histoire Philosophique de l'Hysterie et de l'Hypochondrie, 1833. The womb is, however, no doubt, almost always the "point de depart" of this curious phenomenon, which has given rise to many of the absurd delusions on record, such as women being possessed with the devil, and inwardly tormented and devoured by animals, which have been generated in their bellies, &c.

The abdominal muscles are sometimes the seat of partial convulsions. M. Dubois mentions the case of a young woman, six months pregnant, in whom the parietes of the abdomen contracted with so much violence, that the distended uterus was literally so squeezed fairly into the pelvic cavity, that it could not be felt for the time; and then it would bound forwards, like an elastic ball which has been struck against the ground. Other "bosselures" were seen in the epigastrium, umbilical region, and sides; they seemed to depend upon a spasmodic contraction of the viscera within, as well as of the abdominal parietes.

By far the most important of all the partial puerperal convulsions are those of the uterus itself—they may take place either during gestation, at the time of labour, or after the delivery of the child. M. Menard says that he has known the pregnant uterus assume during convulsions the shape of a calebash (a species of gourd); M. Baudelocque cites a case in which it rose, fell, moved to one side, then to another, with most surprising violence. M. Petit assures us that, in the case which he saw, the convulsions were so strong, that the uterus seemed at every moment to be forced down into the vagina, and that he was obliged to support it with his hand to prevent its protrusion; and M. Pacoud, in his "Compte rendu de la Maternité de Bourg," 1825, relates an example of the womb being affected with actual movements, and with a general violent agitation. The instances we have now recorded are, however, of very rare occurrence; and it is very generally only during the throes of labour, that the uterus is the seat of convulsive movements. The part most commonly affected is the inferior third of the body, and the cervix of the organ; and accoucheurs have remarked, that the period of labour at which the accident usually occurs, is when the head only of the infant has just escaped from the os uteri, while the rest of the body is still within. So strong is the spasmodic constriction, in some cases, that when attempts have been made to deliver with the forceps, the head has actually been torn away from the body; and Dr. Smellie alludes to a case, in which he was obliged to perforate the head and evacuate its contents, before delivery could be effected: M. Menard has related an example, in which the difficulty was still greater—the labour had continued for four days, the head had been torn away by the repeated traction, and even after this, the accoucheur experienced extreme difficulty in bringing down the arms; the upper portion of the cervix uteri was girt round the thorax like an iron brace; and it was only after repeated and fruitless efforts, that at length the hand could get hold of the feet to complete the delivery. The child proved to be ascitic.

Even after the birth of the child, the uterus may be affected with partial or general spasm; and, indeed, that species of retention of the placenta, which our Continental brethren designate "le chatonnement," is very commonly the effect of such a condition. It has been observed that, in such cases, the convulsive movements are usually limited to that part, beneath or nearer to the cervix than the contracted ring; sometimes, however, the upper portion is also involved, and the accoucheur is able to detect a trembling motion or "fremissement" of its anterior parietes.

It is not our intention to enlarge upon the subject of partial or local convulsions occurring in the puerperal state, as they are neither so common, nor nearly so alarming, as when they are more general, and affect the whole muscular system. We have already alluded to the variety of types or characters which the convulsions may assume, and to the division of the generic disease into four species or forms, viz. the hysterical, epileptic, tetanic, and apoplectic, to which some authors have added two others, viz. the cataleptic and choreic. What are the circumstances which induce or favour the occurrence of one or other of these forms, have hitherto not been satisfactorily examined. The constitution and temperament of the patient will necessarily have much influence; so, also, will the period of gestation and of labour, at which the attack supervenes; thus, it has been observed that the apoplectic eclampsia is not nearly so frequent before as it is during the act of accouchement, whereas the very reverse holds true of the hysteric form of the disease; and a similar remark may be made respecting the occurrence of partial and general convulsions—the former are more common before and after, than during labour. In order that we may the better be able to discriminate between the forms or species, it will be useful to keep in mind the most leading and essential features of the generic disease. The attack is very frequently preceded by certain premonitory symptoms, such as flushes of heat about the face, and throbbing of the temples, confusion of the ideas, rambling in the conversation, general restlessness and inquietude, &c.; but sometimes no warning is given, and the paroxysm is most sudden and unexpected: the whole body becomes at once agitated and convulsed—the limbs are bent, extended, twisted, and tossed about with fearful violence—the trunk is thrown spasmodically forwards, then backwards, then dashed from side to side—the features are distorted—every muscle of the face seems in motion, and the eyes roll frightfully about, or appear almost forced from their sockets, giving a most hideous aspect—the tongue is equally affected—a quantity of froth is spurted from the mouth, and the patient grins and gnashes her teeth with seeming horrid rage. The violent action of the diaphragm induces sobbing, and fits of apparent suffocation—the heart labours, and every artery of the body, but more especially the carotids and their branches, throb with prodigious strength, while at the same time the veins are gorged with their dark contents. The stomach, intestines, womb, and bladder all suffer from the general spasm; hence the violent vomitings, the involuntary discharge of the fæces, urine, and often even of the fœtus itself. The duration of this truly frightful state may vary, from five minutes to half, or even a whole hour; and when it ceases, the patient may either become quite rational and quiet, or she may lapse into a comatose or syncopic torpor, which may continue for 12 or 24 hours before the return of consciousness and sensibility. In most cases there is a return of the paroxysm. Drs. Croft and Merriman make the important remark, that the return is often preceded by a remarkable slowness of the pulse, and M. Velpeau's experience confirms the accuracy of this test. When the patient recovers her consciousness, she generally feels exhausted, as if beaten all over, nervously timid, easily alarmed; and she cannot believe the accounts of her attendants, if they are so imprudent as to tell her of what has taken place; even when the child has been expelled, she often seems quite ignorant of it, and can with difficulty be convinced that she has been delivered.

It occasionally happens that certain functions remain paralysed, or perverted, after the fit has ceased; in some cases the sight of one or of both eyes, the hearing, taste, or sense of smell is lost; at other times, the mind exhibits partial or general disturbance of its power.

Such is a rapid sketch of the leading features of puerperal eclampsia; and keeping this in view, as an epitome or portrait of the genus, our readers will be better enabled to appreciate and to compare the chief differences of the species comprehended under it.

1. *The Hysteric.* This form of the disease usually commences by involuntary sobbings, or sighing, by yawning and stretching of the limbs, by a sense of constriction in the throat, so that any efforts to swallow are painful and difficult, by great flatulence in the bowels, and often by a troublesome incontinence of urine.

The extensor muscles are generally more affected with spasm than their antagonists; hence the body is in most cases rigidly stretched back, the arms are repeatedly thrown forwards in front of the neck and chest, as if for the purpose of warding off an injury; but the muscles of the face are not so much distorted in this as in the other forms of the disease. The duration of the paroxysm is seldom long, and it terminates usually by an abundant flow of tears. The consciousness is almost invariably restored immediately after the cessation of the fit; and even although the patient may remain motionless, and does not answer questions, nor even seem to understand them, there is no distinct coma, the breathing being without stertor, and the pupils obedient to the action of light. The pulse is generally small and weak; in short, we may regard such a state as partaking more of the character of syncope than of comatose stupor. We have already remarked that the hysteric eclampsia is more frequent during the first four months of pregnancy than at any subsequent period; it is much less dangerous than the other forms of the disease, and rarely is so severe as to prevent the satisfactory and natural completion of gestation and of delivery.

2. *The Tetanic Eclampsia.* The tonic contractions, in this species, seldom affect the whole of the body, but are usually confined, sometimes to one part, sometimes to another, commencing either in the jaw or in the limbs. The spasms, although certainly differing from what are called the common alternating, or clonic convulsive movements, exhibit distinct remissions:—During their continuance, the patient is ordinarily deprived of all consciousness, and she does not, therefore, experience the agonies of pain which are felt in tetanus, when it occurs idiopathically or after wounds.

3. *Epileptic Eclampsia.* This is the most frequent form of puerperal eclampsia, and to it is the description previously given chiefly applicable. The hysteric, choreic, or tetanic features may characterize the debut of the attack, or accompany and modify it in its progress; but very generally the superadded symptoms of the distention of the face and neck, the cerebral congestion, the frothing at the mouth, and the irregular motions of the tongue and jaw, distinctly announce the prevailing type of the disease. After the fit has ceased the body remains pale; but still a degree of general puffiness and swelling is obvious; for example, if the patient is lying on her side, the lips are often observed to obey their mere weight, and hang down, as if all control over them was lost—hence the saliva keeps dribbling out continually. Although, however, the patient be quite unconscious, and lies in a state of stupor and insensibility, it would be wrong to consider this as actual coma; for her respiration may be calm, and void of all stertorous noise—if moved and addressed loudly, she may open her eyes, and utter some confused and indistinct answer, then relapse into her former apathy, either quietly, or in some degree peevishly, for having been disturbed. This condition of things may last for several hours, and then either give way gradually to returning sensibility, or be succeeded by another paroxysm of convulsions, without any interval of consciousness.

"Epilepsy, properly so called (says M. Velpeau), differs from the epileptic eclampsia, not only, as Mr. Burns affirms, in being dependent upon an organic lesion of the brain, whereas the latter arises from sympathetic irritation, and in being more or less regularly periodic in its returns, as Sauvages has pointed out, but also in its first attacks being always feeble, of short duration, and occurring only at long intervals—in the agitation being less general, the convulsions less multiplied and varied, and in the more speedy return of consciousness and sensibility."

4. *The Apoplectic Eclampsia.* The adjective appellation of this form of the disease points out at once its distinguishing features—the oppression and stupor are more marked, the convulsions more heavy and embarrassed, the respiration stertorous, the pulse slower and fuller, and the pupils less obedient to light than in the other forms. The attack is also usually more gradual, being very often preceded by the signs of cerebral fulness. The muscular system is relaxed, and, as it were, dead—the eyelids, if separated, seem to fall together only by their weight; even the energies of the womb are greatly impaired, and hence the child is seldom expelled during the continuance of the disease. The periods at which the apoplectic eclampsia usually occur are, during labour, especially during its second stage, and also after the delivery. Instances are, however, recorded by Burns, Menard, and others, of its attacks in the early periods of pregnancy.

Under one or other of these four forms now enumerated, every case, perhaps, of puerperal convulsions may be satisfactorily included. It is scarcely necessary to state that the disease is one of most urgent danger; the most unfavourable symptoms are the repeated invasion of the paroxysm at short intervals, and the intervening coma being profound.

Denman has seen it prove fatal in 35 minutes from the first attack; and Schedel, Hamilton, Dewees furnish numerous examples of death, supervening in from 12 to 24 hours. One of the most frequent of the immediate causes of death is sanguineous effusion within the cranium. Rupture of the uterus is an occasional accident.

Even when the patient does recover, it is not uncommon that some distressing results are found to have been induced; thus different forms of insanity, of paralysis, &c. may be left behind.

Madame Lachapelle says that many women who have suffered from eclampsia become affected with inflammatory and congestive diseases; and she instances peritonitis, as one of the most frequent of such accidents. The pathological anatomy of puerperal convulsions is still very obscure. A small quantity of serosity may be found in the ventricles of the brain; its veins and sinuses may be engorged, its membranes and substance may be somewhat redder than is natural; but in numerous cases, even these unsatisfactory lesions, may be entirely absent, or only doubtfully existent.

The heart is sometimes found flaccid and empty; the lungs either congested or unusually pale; a few ounces of yellowish serum are sometimes contained within the thorax or abdomen;—such are the unsatisfactory appearances which Dr. Denman has detailed. Hewson, Hooper, and Ley, each records an example of encephalic sanguineous effusion. M. Cruveilhier, certainly one of the highest authorities on every subject connected with pathological anatomy, assures us, that very often he has not been able to discover a single appreciable lesion; and Mad. Lachapelle has formally announced, that if apoplexy be not coincident with puerperal convulsions, the post-mortem appearances are almost always quite insufficient for the explanation of the symptoms during life. The state of the spinal marrow has probably not been sufficiently often examined to authorise the assertion, that it is as little organically affected, as the brain. It would certainly be interesting to ascertain the condition of the hypogastric nerves, and of the lumbar portion, especially, of the cord.

So much for the pathology of puerperal convulsions. Our knowledge on this subject is exceedingly incomplete, and is to be regretted the more, as it is not improbable that, were it more accurate and precise, the therapeutic indications might be more obvious, and the treatment therefore more successful. Of all the remedies which have been used, blood-letting, copious and repeated, has been most universally confided in; but useful, and absolutely necessary though it may be, we have little doubt but that it has been practised far too indiscriminately and empirically, without regard to the specific characters, and varying circumstances of the cases. M. Velpeau is obliged to confess, that many of the most eminent French accoucheurs are comparatively very unsuccessful in their treatment of eclampsia; and that Mauriçeau and Lachapelle, both of whom employ bleeding to a very large extent, lose at least one half of their patients, while Dr. Merriman, who is much more sparing in the use of the lancet, has asserted that he saves two-thirds of his. Far be it from us to condemn venæsection in most cases; it is very generally necessary, and often quite indispensable, for the salvation of our patient; all that we mean to insist upon is, that our practice should be founded on the scientific principle of studying each particular case, and of adapting our treatment to its special exigencies. One case may be benefited by a single bleeding, and aggravated by a second; while another case requires its repetition, perhaps half a dozen times in the course of 24 hours. As a general rule, we have no hesitation in asserting that bleeding is by far the most potent antispasmodic against puerperal convulsions, and that all the internal remedies which have acquired that title are often not only useless, but positively injurious, if exhibited before blood be withdrawn. The use of them, however, immediately after a copious venæsection, is often most highly successful; and none deserves our confidence so well as camphor in large doses, combined with hyosciamus. The practice which we usually adopt is to give ten grains of camphor, with a drachm of the tincture of hyosciamus, in a little distilled water, every two or three hours; employing, at the same time, a stream of cold water on the shaved head, and applying mustard poultices to the thighs and to the pit of the stomach. Where the excitement is unusually violent, and the patient is of a full and plethoric temperament, we are in the habit of delaying the use of these remedies, (we mean camphor and henbane,) until the energies of the circulatory and nervous systems are brought down by free bleeding, and by the use of the tartar emetic, given in appropriate doses to produce a degree of nausea, or even of retching. One very great advantage obtained from inducing such a state is the general relaxation of every part, including the orifice and neck of the womb, which follows; hence the delivery, whether it be effected altogether by natural, or must be promoted by artificial means, is much more speedy and safe. When once the convulsions have been checked, or considerably reduced in frequency and force, a large dose of calomel, followed speedily by a powerful senna draught, should be given; for in this way, not only is a derivation from the head produced, but the stupifying effect of the antispasmodics is counteracted, and the expulsive action of the womb may be assisted at the same time. Our limits prevent us from any further details; it must be almost unnecessary to state, that in a large number of cases, we are obliged to accelerate the delivery of the child, either with the forceps, or by turning: and that in the event of the convulsions occurring before the period of labour, it becomes a question of high importance, whether we should induce premature labour. No medical man should undertake this last-mentioned operation, without the consent of another professional brother; but when the urgency of the case demands it, all fine-drawn scruples should be laid aside. Ere long, we have no doubt, the practice will be recognised by the French school, although hitherto it has been denounced as a foul and impious heresy. On this, as upon almost every other subject of medical practice, our neighbours are singularly fertile in their inventions; for, not satisfied with the hitherto adopted practice of gently opening the os uteri, and either rupturing the membranes, or separating them cautiously from their attachment to the cervix, they recommend "le debridement du col," or the notching of the cervix uteri with the scalpel, to facilitate its extension. Well might Mauriçeau say that the proposal "ne pouvait etre que le fruit d'un instant de delire." M. Velpeau seems nevertheless to approve of it; but his approval "n'importe;" for he has had no experience of its effects.

Although we have devoted a few pages to the consideration of the theme of our author's work, we have purposely omitted any frequent reference to its contents; for, like many of his other productions, it has been by far too hastily got up, and bears throughout too much the aspect of a mere thesis to stand the ordeal of a rigid criticism.

